# Amoebic liver abscess in a COVID-19 patient: a case report

**DOI:** 10.1186/s12879-021-06819-9

**Published:** 2021-11-04

**Authors:** Andrea L. Maricuto, Viledy L. Velásquez, Jacinto Pineda, David M. Flora-Noda, Isaac Rodríguez, Crismar A. Rodríguez-Inés, Óscar O. Noya-González, Rosa Contreras, Óscar D. Omaña-Ávila, Iván A. Escalante-Pérez, Natasha A. Camejo-Ávila, Nicolle A. Kuffaty-Akkou, Fhabián S. Carrión-Nessi, Martín Carballo, María E. Landaeta, David A. Forero-Peña

**Affiliations:** 1 Infectious Diseases Department, University Hospital of Caracas, Caracas, Venezuela; 2 “Dr. José Antonio O’Daly” Anatomopathological Institute, Central University of Venezuela, Caracas, Venezuela; 3 Radiodiagnosis Department, University Hospital of Caracas, Caracas, Venezuela; 4 Surgery Department, University Hospital of Caracas, Caracas, Venezuela; 5 “Dr. Félix Pifano” Tropical Medicine Institute, Central University of Venezuela, Caracas, Venezuela; 6 “Luis Razetti” School of Medicine, Central University of Venezuela, Caracas, Venezuela; 7 Biomedical Research and Therapeutic Vaccines Institute, Ciudad Bolivar, Venezuela; 8 “Dr. Francisco Battistini Casalta” Health Sciences School, University of Oriente – Bolivar Nucleus, Ciudad Bolivar, Venezuela

**Keywords:** Amoebic liver abscess, *Entamoeba histolytica*, COVID-19, SARS-CoV-2, Venezuela, Case report

## Abstract

**Background:**

Amoebiasis is a parasitic disease caused by *Entamoeba histolytica*, which affects people living in low- and middle-income countries and has intestinal and extraintestinal manifestations. To date, knowledge on coronavirus disease 2019 (COVID-19) coinfection with enteric parasites is limited, and *E. histolytica* coinfection has not been previously described. Here we present the case of a patient with COVID-19 who, during hospitalisation, presented a clinical picture consistent with an amoebic liver abscess (ALA).

**Case presentation:**

A 54-year-old man, admitted as a suspected case of COVID-19, presented to our hospital with dyspnoea, malaise, fever and hypoxaemia. A nasopharyngeal swab was positive for severe acute respiratory syndrome coronavirus 2 (SARS-CoV-2) by reverse-transcription polymerase chain reaction. After 7 days, he developed diarrhoea, choluria and dysentery. An abdominal ultrasound showed a lesion compatible with a liver abscess; stool examination revealed *E. histolytica* trophozoites, and additional serology for *E. histolytica* was positive. After 12 days of treatment with metronidazole, ceftazidime and nitazoxanide, the patient reported acute abdominal pain, and an ultrasound examination revealed free liquid in the abdominal cavity. An emergency exploratory laparotomy was performed, finding 3000 mL of a thick fluid described as “anchovy paste”. Computed tomography scan revealed a second abscess. He ended up receiving 21 days of antibiotic treatment and was discharged with satisfactory improvement.

**Conclusion:**

Here we present, to the best of our knowledge, the first report of ALA and COVID-19 co-presenting. Based on their pathophysiological similarities, coinfection with SARS-CoV-2 and *E. histolytica* could change the patient’s clinical course; however, larger studies are needed to fully understand the interaction between these pathogens.

## Background

Coronavirus disease 2019 (COVID-19), caused by severe acute respiratory syndrome coronavirus 2 (SARS-CoV-2), has become a public health problem worldwide [1]. The lung is considered the main organ affected by COVID-19; however, extrapulmonary symptoms may also manifest during the disease. SARS-CoV-2 was detected in the stool of the first reported case of COVID-19 in the United States, which also presented digestive symptoms, prompting interest in the gastrointestinal manifestations of COVID-19 [2]. Gastrointestinal and hepatic presentations are not rare; diarrhoea and mild elevation of liver enzymes are the most common characteristics of COVID‐19, in the absence of pre-existing diseases [3]. Recent studies on COVID-19 have shown a high incidence of liver injury ranging from 14.8 to 53%, mainly indicated by abnormal alanine transaminase/aspartate transaminase levels accompanied by slightly elevated bilirubin levels. The proportion of patients who develop liver injury in severe COVID-19 seems to be higher than in mild cases [4–7]. SARS-CoV-2 uses angiotensin-converting enzyme 2 (ACE2) as its entry receptor [8], which is expressed in liver and bile duct cells [9, 10], suggesting direct liver injury in patients with COVID-19.

There is limited knowledge on the prevalence of coinfections among patients with COVID-19, especially in organs and systems other than the respiratory tract. Intestinal parasitic infections affect more than 2 billion people worldwide, with disproportionately high prevalence rates in low- and middle-income countries [11]. Amoebiasis is a parasitic disease caused by *Entamoeba histolytica*, and has intestinal and extraintestinal manifestations. Amoebic liver abscess (ALA) is the most common extraintestinal manifestation in any setting, including Venezuela [12]. Coinfection with SARS-CoV-2 could change the clinical course of the patient; however, this coinfection has not been previously described. Here we present the case of a patient with COVID-19 who, during hospitalisation, presented a clinical picture consistent with an ALA.

## Case presentation

**Fig. 1:**
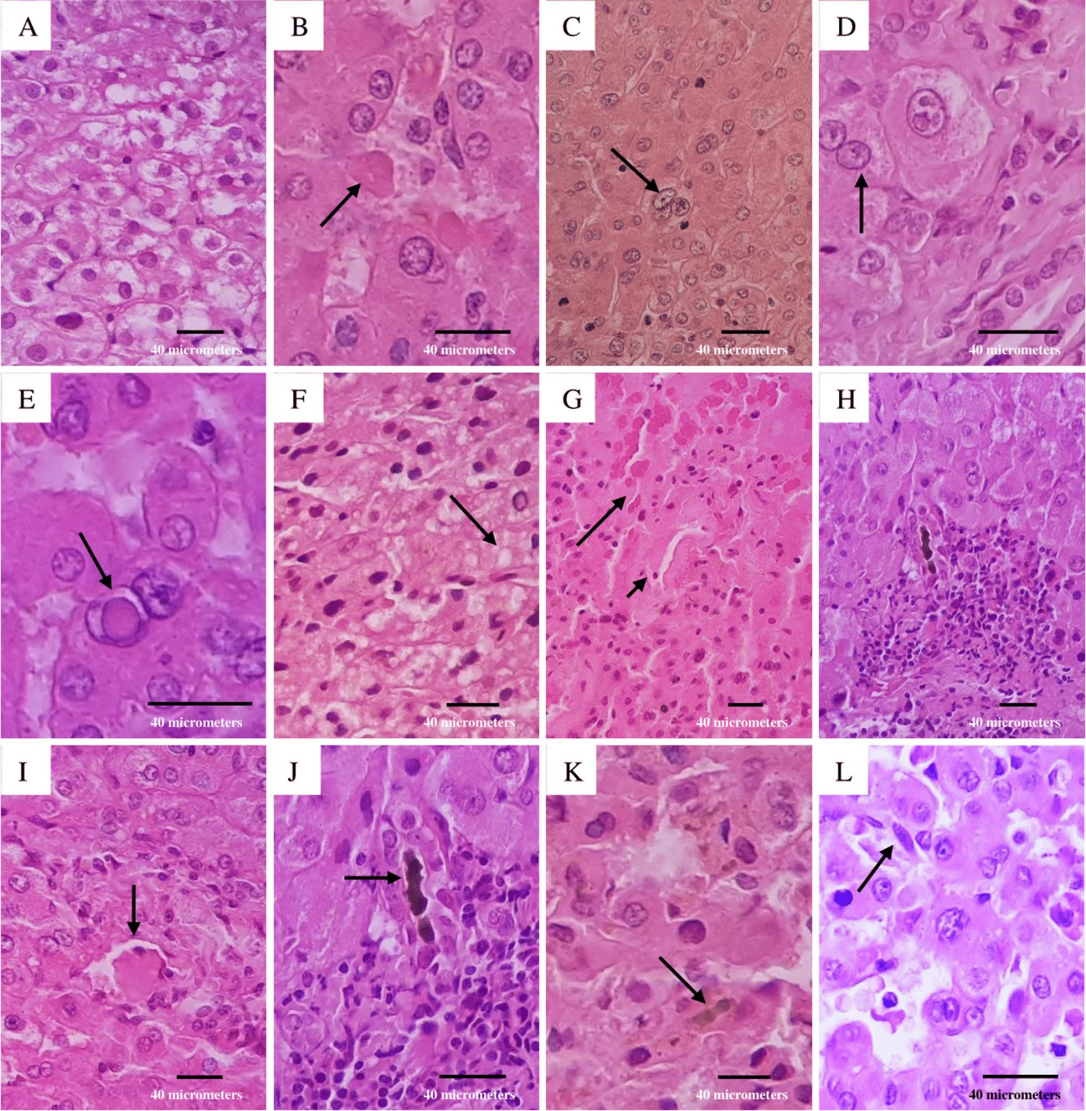
Liver biopsy. **A** Swelling of hepatocytes with increased and pale cytoplasm; **B** eosinophilic round body (arrow); **C** binucleated hepatocytes (arrow); **D** trinucleated hepatocytes (arrow); **E** homogeneous clearing of hepatocyte nuclei (arrow); **F** multiple tiny intracytoplasmic fat droplets that do not displace the nucleus (arrow); **G** necrotic hepatocytes (big arrow) around the central vein (small arrow); **H** inflammatory infiltrate composed by lymphocytes; **I** large fibrin thrombus in portal vein branch (arrow); **J** bile plug in the bile duct (arrow); **K** bile plug in the bile canaliculi (arrow); **L** Kupffer cells proliferate and enlarge in response to hepatocyte damage (arrow). The microscope used was a Leica DM500 (Leica Microsystems Inc., IL, United States) and the image was captured with a Samsung Galaxy M20 smartphone (Samsung Electronics, Seoul, South Korea). Images were acquired at a resolution of 20 megapixels and edited with the phone’s own software, then processed using Microsoft^®^ Excel^®^ version 2019 (Microsoft, WA, United States)

**Fig. 2:**
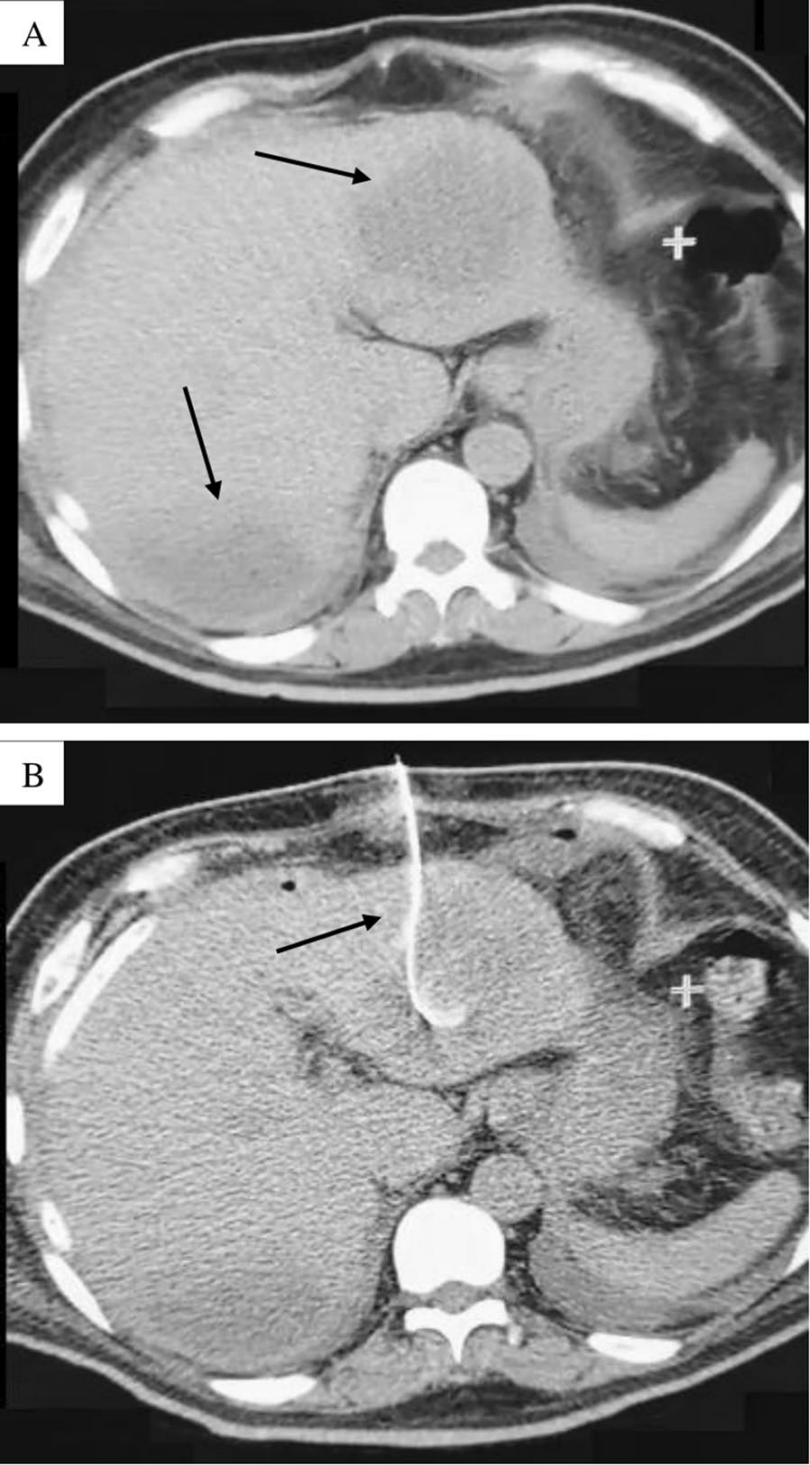
Computed tomography. **A** Axial section of abdominal computed tomography scan without contrast administration showing an enlarged liver (caudal skull diameter: 18 cm), at least two images were located in segments II and VIII of rounded morphology, regular edges, hypodense (Hounsfield Units: 20), the largest with 150 cc volume, likely related to liver abscess; **B** axial section of abdominal computed tomography scan without oral or intravenous contrast administration, showing the placement of a catheter for drainage of the liver abscess located in segment II

A 54-year-old man presented to the Infectious Diseases Department of the University Hospital of Caracas with dyspnoea, malaise and fever over 6 days; he was admitted as a suspected case of COVID-19 due to hypoxaemia (oxygen saturation < 90%) with no significant medical history. On admission, blood test showed neutrophilia (8.05 × 10^3^/μL), lymphopenia (0.88 × 10^3^/μL), mild transaminases elevation (aspartate aminotransferase: 40 U/L; alanine aminotransferase: 69 U/L), bilirubin levels within normal range (total bilirubin: 0.5 mg/dL; indirect bilirubin: 0.3 mg/dL; direct bilirubin: 0.2 mg/dL), and inflammatory markers elevation (lactate dehydrogenase: 394 U/L; C-reactive protein: 9.1 mg/L; erythrocyte sedimentation rate: 55 mm/h). Human immunodeficiency virus serology was performed according to the Infectious Diseases Department protocol, with a non-reactive result. A nasopharyngeal swab was positive for SARS-CoV-2 by reverse-transcription polymerase chain reaction. After 7 days, he presented diarrhoea, choluria and then dysentery; subsequent liver function tests revealed transaminases limit values (aspartate aminotransferase: 31.6 U/L; alanine aminotransferase: 45.8 U/L), and mild indirect bilirubin elevation (total bilirubin: 1.16 mg/dL; indirect bilirubin: 0.74 mg/dL; direct bilirubin: 0.42 mg/dL). An abdominal ultrasound showed an image compatible with an ALA; stool examination revealed *E. histolytica* trophozoites. Serological diagnosis was performed by enzyme-linked immunosorbent assay, obtaining an optical density of 0.325 (our institution’s cut-off point using healthy patient sera was 0.215). Immediately, treatment with metronidazole, ceftazidime and nitazoxanide was started. Although the patient presented clinical improvement, on day 12 of treatment the patient reported acute abdominal pain, and an ultrasound examination revealed free liquid in the abdominal cavity. An emergency exploratory laparotomy was performed, finding 3000 mL of a thick fluid described as “anchovy paste”. Liver biopsy showed hepatocytes with ballooning degeneration (Fig. 1A), acidophilic bodies (Fig. 1B), binucleated and trinucleated hepatocytes (Fig. 1C, D), glycogenic intranuclear vacuolisation (Fig. 1E), moderate microvesicular steatosis (Fig. 1F), necrotic hepatocytes around the central vein (Fig. 1G), moderate inflammation in the portal tracts with occasional lymphocytic infiltrate (Fig. 1H), large fibrin thrombus in the portal vein branch (Fig. 1I), and ductular and intracanalicular cholestasis (Fig. 1J, K). Furthermore, Kupffer cells were hypertrophic (Fig. 1L); there was no interphase hepatitis. Computed tomography scan revealed a second abscess (Fig. 2A), which was drained by percutaneous drainage (Fig. 2B). The patient ended up receiving 21 days of antibiotic treatment and was discharged with satisfactory improvement.

## Discussion and conclusions

Gastrointestinal symptoms are common in patients with COVID-19, but the prevalence of coinfection with enteric pathogens is unknown. Recently, a retrospective study concluded that patients with COVID-19 were less likely to test positive for coinfection with typical gastrointestinal pathogens (except with *Clostridium difficile*) compared to those without COVID-19 [13]. In Venezuela, the prevalence of *E. histolytica*/*E. dispar* infection varies from one region to another (6.8–42%), mainly affecting school-age children from Zulia, Falcón, Táchira, Lara, Aragua and Sucre states [12, 14]; however, the epidemiology of this parasitosis in the Capital District (Caracas) is unknown. Here we present the case of a patient with confirmed COVID-19 who, during hospitalisation, presented a clinical picture consistent with an ALA, identifying *E. histolytica* as the aetiological agent through a stool examination, and, secondly, by the frank seropositivity of an “in-house” ELISA; furthermore, the evidence of a typical image in the liver parenchyma with liquid collection, negative for bacterial infection, supported this idea. However, in this case it was not possible to identify amoebic trophozoites and cysts, probably due to the location of the sample taken, as the parasites are predominantly located around the healthy margins of liver abscesses, where they feed on cytolytised hepatocytes [15].

This case occurred simultaneously with the identification of an in-hospital amoebiasis flare-up caused by hospital kitchen staff infected by this parasite, where 37 persons directly involved in food preparation were evaluated and intestinal amoebiasis was documented in four of them (10.8%) (unpublished data). Considering the faecal-oral transmission of this parasitosis and its incubation time, the patient could acquire the disease by consuming contaminated water or food before admission to the hospital.

*Entamoeba histolytica* infection can be asymptomatic or symptomatic, and can lead to tissue invasion. The most common invasive extraintestinal manifestation is ALA: the parasite reaches the liver via the portal circulation. Usually, in this presentation, there is no evidence of previous colitis, suggesting an asymptomatic infection or confined to the right side of the colon [16]. Persistence, establishment and consequent clinical presentations of infection depend on the pathological characteristics of the protozoan, but it is also affected by the host immune response. ALA occurs in a minority of patients with amoebiasis (3–9%) [16], and complications such as abscess rupture (2.5–22%) [17, 18] and peritonitis (2.4–13%) may develop, associated with higher mortality (40–50%) [17, 19]. ALA diagnosis is performed by clinical history, ultrasound (sensitivity varies as it is an operator-dependent study) [20, 21] or computed tomography scan (higher sensitivity than ultrasound) [22] in combination with a confirmatory laboratory study, such as enzyme-linked immunosorbent assay due to its high sensitivity and specificity [23]. In endemic areas, the use of serological tests based on *E. histolytica* recombinant antigens is preferable, if available, as it only detects active infections [24]. Although 70–80% of ALA cases have only one abscess mainly located in the right lobe of the liver [25], there seems to be an increase of the incidence of multiple ALA due to the improved imaging studies [26]. Coinfection with ALA and gram-negative bacteria, mainly *Klebsiella pneumoniae* and *Escherichia coli*, is commonly found among cases of multiple abscesses [26, 27], although cases of simultaneous infection with hepatotropic virus are anecdotal [28].

ACE2 receptors have been identified in the gastrointestinal tract and liver, which are highly expressed in the endothelial layer of small blood vessels, but not in the sinusoidal endothelium. Recent studies have also found higher ACE2 receptor expression in cholangiocytes than in hepatocytes; in fact, ACE2 levels in cholangiocytes are similar to those in lung type-2 alveolar cells, suggesting that the liver may be a potential target of SARS-CoV-2 [29]. Moreover, elevated prostaglandin E2 (PGE2) levels have been associated with ALA formation due to an immunosuppressive effect of PGE2 [30]. We can hypothesise that, in our patient, elevated PGE2 levels are secondary to both direct stimulation of cyclooxygenase-2 (COX-2) by SARS-CoV2-2 [31] and presence of a COX-2-like protein present in *E. histolytica* trophozoites [30], which could influence the clinical course; however, the available knowledge is still insufficient to correlate them with each other.

In this case, the patient required an exploratory laparotomy which showed secondary peritonitis due to ALA rupture. There is no consensus on the optimal treatment of complicated ALA with diffuse peritonitis; however, percutaneous drainage appears to be the preferred standard treatment in complicated ALA, and evidence suggests that drainage plus antimicrobial therapy (amoebicidal agent) has an excellent outcome [19]. In this case, despite empirical antimicrobial therapy, the patient required an exploratory laparotomy which showed secondary peritonitis due to rupture of the ALA. Combination antimicrobial therapy was maintained as the patient’s condition deteriorated and toxaemia impressed prior to surgery. In addition, adequate samples were not taken before starting empirical antibiotic treatment. Histology showed hepatocytes with ballooning degeneration and acidophilic bodies, consistent with another study [32]. Binuclear and multinuclear hepatocytes were identified, similar to the findings of Wang et al. [33], who report that this morphological phenomenon may be attributed to reactive changes. Few hepatocytes showed glycogenic intranuclear vacuolisation, a finding that contrasts with that found by Pessolani et al. [34], who described many hepatocytes with this finding. In a series of 40 cases, Lagana et al. [35] found macrovesicular steatosis in all patients, whereas microvesicular steatosis was found in our patient. It is well known that drugs, infections, malnutrition and various other insults cause fatty liver [35].

Necrotic hepatocytes and portal inflammation were consistent in the literature consulted. Schmit et al. [36] observed necrosis in zone III in most cases, similar to that found in our patient. Thrombotic phenomenon was observed in our case. The mechanism of this prothrombotic milieu remains to be determined; however, this morphological finding suggest a profound platelet response in COVID-19 that may be responsible, at least in part, for multiple organ dysfunction [37]. An investigation by Diaz et al. [38] showed that this phenomenon may be due to an endothelial dysfunction, a procoagulant state and/or a direct vascular injury of the disease. Ductular and bile canaliculus cholestasis suggest sepsis and functional cholangiocytes injure [35], respectively, a morphological manifestation observed in our patient. Another morphological phenomenon observed was Kupffer cells hypertrophy and hyperplasia, findings similar to those reported by Diaz et al. [38]; however, the pathophysiological mechanism of this morphological manifestation is still unknown.

Although gastrointestinal and liver involvement by SARS-CoV-2 has been described, there is no demonstration that COVID-19 may predispose to other gastrointestinal involvement. Conversely, a recent study found that coinfection with parasitic diseases appears to be associated with reduced severity by COVID-19, suggesting that parasite-driven immunomodulatory responses may mute the hyperinflammation associated with severe COVID-19 [39]. To our knowledge, this is the first report of simultaneous presentation of ALA and COVID-19. In our case, it is likely that there is no causal relationship; however, the likelihood of coinfection is highlighted in regions with a high incidence of intestinal amoebiasis, which should be diagnosed and treated early. Based on their pathophysiological similarities, coinfection with SARS-CoV-2 and *E. histolytica* could change the patient’s clinical course; however, larger studies are needed to fully understand the interaction between these pathogens.

## Data Availability

All data and materials in this article are included in the manuscript.

## Abbreviations

COVID-19: Coronavirus disease 2019
SARS-CoV-2: Severe acute respiratory syndrome coronavirus 2
ACE2: Angiotensin-converting enzyme 2
ALA: Amoebic liver abscess
PGE2: Prostaglandin E2
COX-2: Cyclooxygenase-2
